# Inhibition of VEGF_165_/VEGFR2-dependent signaling by LECT2 suppresses hepatocellular carcinoma angiogenesis

**DOI:** 10.1038/srep31398

**Published:** 2016-08-10

**Authors:** Chi-Kuan Chen, Wen-Hsuan Yu, Tsu-Yao Cheng, Min-Wei Chen, Chia-Yi Su, Yi-Chieh Yang, Tsang-Chih Kuo, Ming-Tsan Lin, Ya-Chi Huang, Michael Hsiao, Kuo-Tai Hua, Mien-Chie Hung, Min-Liang Kuo

**Affiliations:** 1 Genomics Research Center, Academia Sinica, Taipei, Taiwan; 2 Graduate Institute of Toxicology, College of Medicine, National Taiwan University, Taipei, Taiwan; 3 Department of Molecular and Cellular Oncology, The University of Texas MD Anderson Cancer Center, Houston, TX, USA; 4 The University of Texas Graduate School of Biomedical Sciences at Houston, Houston TX, USA; 5 Center for Molecular Medicine and Graduate Institute of Cancer Biology, China Medical University, Taichung, Taiwan; 6 Department of Laboratory Medicine, National Taiwan University Hospital, Taipei, Taiwan; 7 Department of Internal Medicine, National Taiwan University Hospital, Taipei, Taiwan; 8 Department of Oncology, National Taiwan University Hospital, Taipei, Taiwan; 9 Graduate Institute of Oncology, College of Medicine, National Taiwan University, Taipei, Taiwan; 10 Institute of Biochemical Sciences, College of Life Science, National Taiwan University, Taipei, Taiwan; 11 Department of Surgery, National Taiwan University Hospital, and National Taiwan University College of Medicine, Taipei, Taiwan; 12 Department of Primary Care Medicine, National Taiwan University Hospital and National Taiwan University College of Medicine, Taipei, Taiwan; 13 Graduate Institute of Microbiology, College of Medicine, National Taiwan University, Taipei, Taiwan

## Abstract

Hepatocellular carcinoma (HCC) relies on angiogenesis for growth and metastasis. Leukocyte cell-derived chemotaxin 2 (LECT2) is a cytokine and preferentially expressed in the liver. Previous studies have found that LECT2 targets to both immune and tumor cells to suppress HCC development and vascular invasion. Although LECT2 did not affect HCC cells growth *in vitro*, it still suppressed HCC xenografts growth in immune-deficient mice, suggesting other cells such as stroma cells may also be targeted by LECT2. Here, we sought to determine the role of LECT2 in tumor angiogenesis in HCC patients. We found that LECT2 expression inhibited tumor growth via angiogenesis in the HCC xenograft model. Specifically, we demonstrated that recombinant human LECT2 protein selectively suppressed vascular endothelial growth factor (VEGF)_165_-induced endothelial cell proliferation, migration, and tube formation *in vitro* and *in vivo*. Mechanistically, LECT2 reduced VEGF receptor 2 tyrosine phosphorylation and its downstream extracellular signal-regulated kinase and AKT phosphorylation. Furthermore, LECT2 gene expression correlated negatively with angiogenesis in HCC patients. Taken together, our findings demonstrate that LECT2 inhibits VEGF_165_-induced HCC angiogenesis through directly binding to VEGFR2 and has broad applications in treating VEGF-mediated solid tumors.

Hepatocellular carcinoma (HCC) is the most common primary liver cancer and the third leading cause of cancer deaths worldwide. Unfortunately, the therapeutic options for advanced HCC are limited, and the disease often recurs even after aggressive local treatment^1^,^2^. HCC is known to be one of the most vascular solid tumors, in which angiogenesis plays an important role in tumor progression and contributes to high recurrence and poor survival rates^3^. Various growth factors regulate angiogenesis of HCC, such as vascular endothelial growth factor (VEGF), platelet-derived growth factor (PDGF), basic fibroblast growth factor (bFGF), and their related pathways^4^. VEGF family members are the major growth factors that regulate HCC progression^5^. This family consists at least five isoforms, and researchers initially discovered the most prominent VEGF-A isoform, VEGF_165_, as a tumor-secreted protein that increases vascular permeability^6^. VEGF family members exert their activities by binding to VEGF receptors (VEGFRs) 1, 2, and 3. VEGFR2 (also known as KDR or FLK1) is the primary receptor mediating the angiogenic activity of VEGF in distinct signal transduction pathways, which regulate endothelial cell proliferation, migration, differentiation, and tube formation^7^.

Investigators originally identified leukocyte cell-derived chemotaxin 2 (LECT2) as a chemotactic factor for neutrophils, and it stimulates the growth of chondrocytes and osteoblasts^8^,^9^. Subsequent isolation of LECT2-coding cDNA suggested that it is predominantly expressed in the liver^10^. LECT2 is a 16-kDa secreted protein containing 133 amino acids and 3 intramolecular disulfide bonds^11^. The human LECT2 gene is mapped to chromosome 5q31.1-q32, a cluster harboring several genes encoding for immunomodulatory cytokines such as interleukin (IL)−3, −4 and −5 and granulocyte macrophage-colony stimulating factor^12^. Consistent with the originally described immunomodulatory effects of LECT2, the authors reported that livers in LECT2-knockout mice had increased numbers of invariant natural killer T cells together with excessive IL-4 and Fas ligand expression, suggesting an anti-inflammatory action of LECT2^12^. Moreover, dysregulation of LECT2 can be found in hepatic tissue under a variety of pathological conditions, including acute liver failure, liver regeneration after partial hepatectomy, and concanavalin A-induced liver injury^13^,^14^,^15^. Recently, researchers found that LECT2 participates in the HCC developmental process^16^,^17^. Specifically, LECT2 expression was highly correlated with improved prognosis for and prolonged survival of HCC^16^. We previously identified the hepatocyte growth factor (HGF) receptor MET as an important target of LECT2 in HCC cells using liquid chromatography tandem-mass spectrometry and a receptor tyrosine kinase (RTK) array. LECT2 bound directly to the α chain of the MET extracellular domain and inhibited MET signaling by recruiting PTP1B to c-terminal of MET^17^. By using a NSG (NOD scid gamma; NOD.Cg-Prkdc^scid^ Il2rg^tm1Wjl^/SzJ) immunocompromised mouse model, in which almost all of the immune cells are lost, we excluded the potential immunomodulatory effects of LECT2 on tumor inhibition.

Collectively, clinical and mechanistic findings from our own and other studies suggest that LECT2 is an important regulator of tumor growth during HCC development and progression. A secretary protein like LECT2 may also affect stromal cells in tumors. In this study, we found that LECT2 suppressed tumor growth *in vivo* without affecting cancer cell proliferation *in vitro*. On the basis of these findings, we hypothesized that LECT2 not only suppresses vascular invasion and metastasis of HCC cells but also inhibits tumor growth by targeting stromal cells. We first demonstrated that LECT2 suppressed HCC growth by inhibiting tumor angiogenesis *in vivo*. We then elucidated the antiangiogenic effect and underlying mechanisms of tumor-stroma interaction by LECT2. Finally, we evaluated the correlation of LECT2 expression with tumor angiogenesis in HCC patients.

## Materials and Methods

### Cell culture

Human umbilical vein endothelial cells (HUVECs) were isolated from fresh human umbilical cords as described previously^18^ and cultured in EGM-2 medium (Lonza). HUVECs from two or more donors were pooled together to prevent genetic variations caused by sampling of the cells. HUVECs were synchronized in the G_0_-G_1_ phase by serum starvation for 12 h in M199 medium (Gibco) containing 1% fetal bovine serum (Gibco) and 0.1% bovine serum albumin (Sigma) before stimulation with the indicated angiogenic factors. Moreover, hepatoma cell lines SK-Hep1, PLC/PRF5 and BNL 1ME A.7R.1 [BNL] were obtained from ATCC, and Huh 7 cell line was obtained from JCRB. HCC36 was established from HCC tissues from a Taiwanese patient^19^. All cells were routinely authenticated on the basis of morphologic and growth characteristics as well as by STR analysis and confirmed to be free of mycoplasma. Cells were grown in Dulbecco’s modified Eagle’s medium (Gibco) with 10% fetal bovine serum (Gibco) at 37 °C in a humidified atmosphere of 5% CO_2_/95% air. Cells were cultured according to the supplier’s recommendations.

### Tube formation assay

HUVECs were seeded on Matrigel, differentiated and formed capillary-like tube structures. Tube formation on Matrigel requires cell-matrix interaction and cellular communication and motility^20^. To examine the effect of recombinant LECT2 (rLECT2) protein expression on angiogenesis *in vitro*, HUVECs were seeded in 24-well culture plates (4.5 × 10^4^ cells/well) precoated with Matrigel and exposed to different concentrations of rLECT2 protein (0, 1.25, 2.50, or 5.00 nM) or recombinant Fc (rFc) protein (R & D) as a control for 6 h. Tube formation was visualized under an inverted microscope. An enclosed network of tube structures in six randomly chosen fields was scored under the microscope. In some tube formation experiments, HUVECs were exposed to angiogenic factors or conditioned media of cancer cell lines for 6 h in the presence or absence of 5 nM rLECT2 protein.

### Wound healing assay

HUVECs were cultured on 24-well plates (7 × 10^4^ cells/well) in EGM-2 medium. After 24 h, the cells were supplemented in starvation medium and scratched with a blue pipette tip to obtain a monolayer culture with a space without cells. Media and dislodged cells were aspirated from the plates, and fresh medium was added to the plates along with VEGF_165_ or rLECT2 protein at various concentrations at 37 °C for 14 h. The migrated cells were photographed at 0 and 14 h using an inverted phase-contrast microscope, and the migrating cells were measured in five randomly chosen fields. The cell migration from the edge of the injured monolayer was quantified by measuring the distance from the wound edges.

### Histology and immunohistochemistry

Tumor samples obtained from HCC patients or mice were harvested and fixed in formalin for paraffin sectioning. Tumor sections used for immunostaining were obtained from formalin-fixed, paraffin-embedded primary tumors removed from HCC patients or frozen primary tumors generated in mice via subcutaneous injection of HCC cell lines. The samples were stained with the primary antibodies CD34 (Dako) or CD31 (Dako) overnight at 4 °C. Bound antibodies were detected in the samples using an ABC kit (Vector Laboratories). Slides containing tumor sections were stained with diaminobenzidine, washed, counterstained with Delafield’s hematoxylin, dehydrated, treated with xylene, and mounted. To quantify the angiogenesis in the samples, MVD was determined by staining tissue sections immunohistochemically for the pan-endothelial cell antigen. Three highly vascularized areas per tumor were then evaluated at high magnification (200×). The total number of microvessels was determined for each area, and the average number was documented for each tumor.

### Xenograft mouse model

Female mice were randomly divided into groups of five mice per group. SK-Hep1/control and SK-Hep1/LECT2 cells (5 × 10^5^ cells) were injected subcutaneously into the right flank of the NSG (NOD scid gamma; NOD.Cg-Prkdc^scid^ Il2rg^tm1Wjl^/SzJ) mice (4–5 weeks). BNL/control and BNL/LECT2 cells (5 × 10^5^ cells) were injected subcutaneously into BALB/C mice (4–5 weeks). The tumor sizes were determined by Vernier caliper measurements and calculated as length × width × 1/2 width. Twenty-four days after the injection, the subcutaneous tumors were excised, weighed, photographed, and a portion of each was placed in 10% formalin for paraffin embedding in preparation for subsequent immunohistochemical analysis.

### Statistical analysis

Each experiment in this study was performed in triplicate, and all experiments were repeated at least three times on different occasions. The data are presented as the mean ± SD. The Student *t*-test was used to compare data among groups. All statistical tests included two-way analysis of variance. Statistical significance was assumed at *P* values less than 0.05.

### Study approval

The experimental protocols for animal care and use were done in accordance with a protocol approved by the National Taiwan University College of Medicine and National Taiwan University College of Public Health institutional animal care and use committees. All animal experiments were performed according to the guidelines and approval of the institutional animal care committee. The human study protocols were also reviewed and approved by the National Taiwan University College of Medicine and National Taiwan University Hospital. All of the tissue and samples were collected at the National Taiwan University Hospital following approval by the Institutional Review Boards and written informed consent. The projects are conducted in accordance with the IRB’s requirements.

## Results

### LECT2 suppresses tumor growth and inhibits tumor angiogenesis

**Figure 1:**
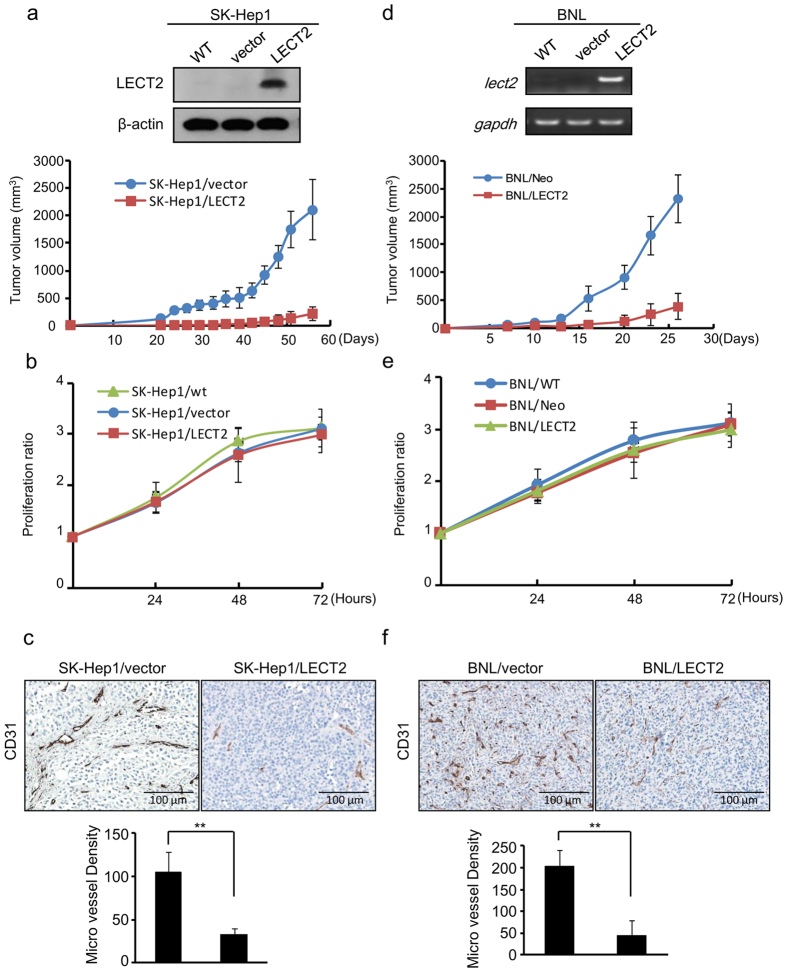
Ectopic LECT2 expression inhibits tumor growth and angiogenesis in an HCC xenograft model. (**a**) Top, analysis of stable expression of LECT2 protein in SK-Hep1 cells by immunoblotting. Bottom, tumor volume was measured by using a two-dimensional caliper at regular intervals in NSG mice inoculated subcutaneously with control or LECT2-expressing SK-Hep1 cells. (**b**) The proliferation ratios of SK-Hep1 cells as determined using an MTT assay for 3 days. Each data point is representative of three independent experiments and presented as the mean ± SD. (**c**) The effects of LECT2 expression on tumor angiogenesis and growth in a xenograft mouse model of HCC. Top, sections of tumors obtained from mice were stained with the specific murine blood vessel marker CD31. Bottom, quantitation of MVD in the xenograft tumors obtained from mice. (**d**) Top, analysis of *lect2* gene expression in stable BNL cells by reverse transcription-polymerase chain reaction. Bottom, tumor volume was measured by using a two-dimensional caliper at regular intervals in BALB/C mice inoculated subcutaneously with control or *lect2*-expressing BNL cells. (**e**) The proliferation ratios of BNL cells as determined using an MTT assay for 3 days. (**f**) The effects of *lect2* expression on tumor angiogenesis and growth in a xenograft mouse model of HCC. Top, sections of tumors obtained from mice were stained with CD31. Bottom, quantitation of MVD in the xenograft tumors obtained from mice.

To determine whether LECT2 affects tumor growth, we used an immunodeficient NSG mouse model of HCC subcutaneously injected with LECT2-overexpressing SK-Hep1 (SK-Hep1/LECT2) cells (Fig. 1a). We first detected palpable tumors in some of the mice by 10 days after cell injection. After 32 days, the mean tumor volumes in mice injected with control SK-Hep1 cells were markedly larger than those in mice injected with SK-Hep1/LECT2 cells (Fig. 1a, bottom). In addition, the incidence of control SK-Hep1 tumors was higher than that of SK-Hep1/LECT2 tumors (data not shown). However, the *in vitro* proliferation properties of the transfectants were not affected by LECT2 expression (Fig. 1b). We stained sections of tumors obtained from the mice with CD31 (PECAM-1; Fig. 1c) and found that the microvessel density (MVD) was markedly lower in the xenograft tumors from the SK-Hep1/LECT2 group than in those from the control group. We performed the same experiment using a BALB/C syngeneic mouse model with chemically transformed BNL murine liver cancer cells and observed results similar to those for SK-Hep1 xenografts model (Fig. 1d–f). These data suggested that ectopic expression of LECT2 diminishes tumor growth likely via inhibition of tumor angiogenesis.

### Secreted LECT2 protein inhibits the angiogenic effect of HUVECs *in vitro*

**Figure 2:**
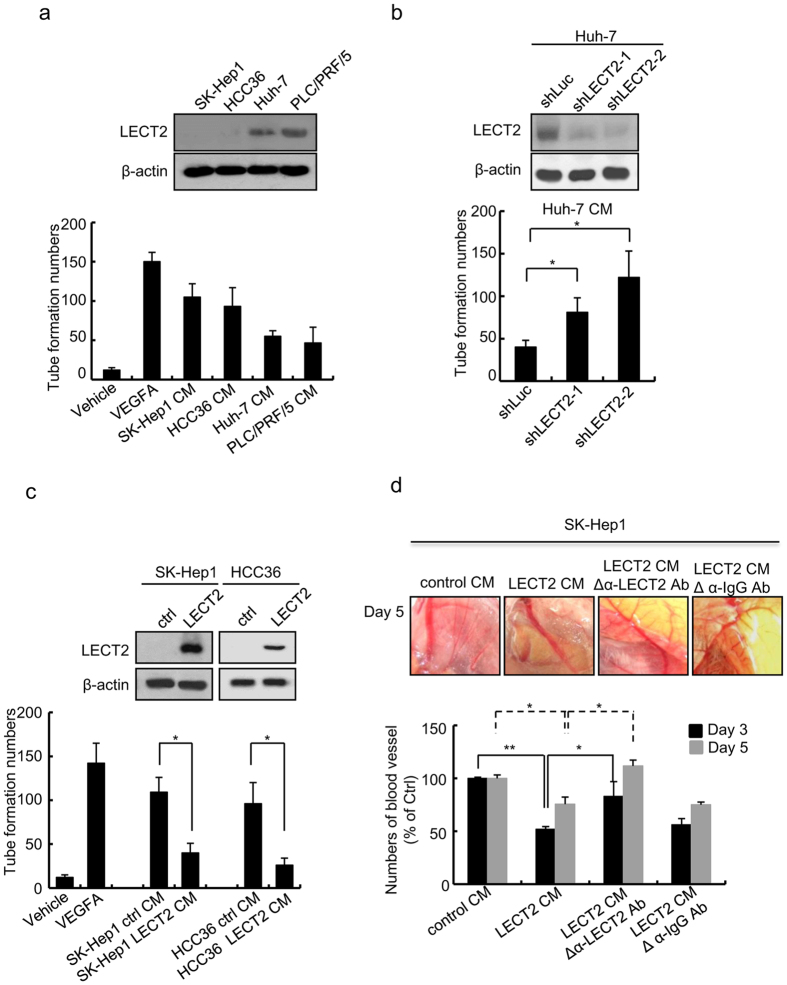
Secreted LECT2 protein inhibits the angiogenic effect in HUVECs. (**a**) Immunoblot of the LECT2 protein expression levels in different HCC cell lines. HUVECs were seeded onto a Matrigel layer in a 24-well plate and treated with the CM from different HCC cell lines in a tube formation assay. Treatment with a vehicle and VEGF_165_ was used as negative and positive control, respectively. The tubular length of HUVEC was quantified using the Image-Pro Plus software program (version 4.5). (**b,c**) The tube formation abilities of HUVEC as determined by using CM from (**b**) LECT2-knockdown Huh7 cells and (**c**) LECT2-overexpressing SK-Hep1 and HCC36 cells. (**d**) Vessel growth in *ex vivo* CAMs of 9-day-old chick embryos incubated with the indicated CM from control and LECT2-expressing SK-Hep1 cells. Vessel growth was assessed by measuring vessel lengths 3 days (day 12) and 5 days (day 14) after inoculation.

Next, we performed a tube formation assay with HUVECs to determine whether secreted LECT2 protein may inhibit HCC angiogenesis. We first collected the conditioned medium (CM) from SK-Hep1, HCC36, Huh7, and PLC/PRF/5 cells and subjected the medium to an *in vitro* tube formation assay with HUVECs. The tube formation ability decreased in high LECT2-expressing CM from Huh7 and PLC/PRF/5 cells but increased in low LECT2-expressing CM from SK-Hep1 and HCC36 cells (Fig. 2a). In addition, the tube formation ability in the CM from LECT2-knockdown Huh7 cells was greater than that in the control CM (Fig. 2b). In contrast, the tube formation ability was lower in the CM from LECT2-overexpressing SK-Hep1 and HCC36 cells than that in the control CM (Fig. 2c).

We further performed an *ex vivo* chicken embryo CAM assay to validate the antiangiogenic effect of LECT2 (Fig. 2d). We incubated CAMs from 9-day-old chick embryos with the CM from SK-Hep1 cells with or without LECT2 overexpression. The results indicated that LECT2-expressing CM markedly decreased the capillary bed area of the chorioallantois on each CAM in comparison to the control CM (Fig. 2d). Next, we used an anti-LECT2 antibody to deplete LECT2 protein in the CM before application to CAMs. The antiangiogenic effects were diminished in LECT2-expressing CM pretreated with the LECT2 antibody but not normal IgG. These results suggest that LECT2 protein acts as an antiangiogenic factor in CM (Fig. 2d).

### rLECT2 protein inhibits HUVEC migration and tube formation induced by angiogenic factors

**Figure 3:**
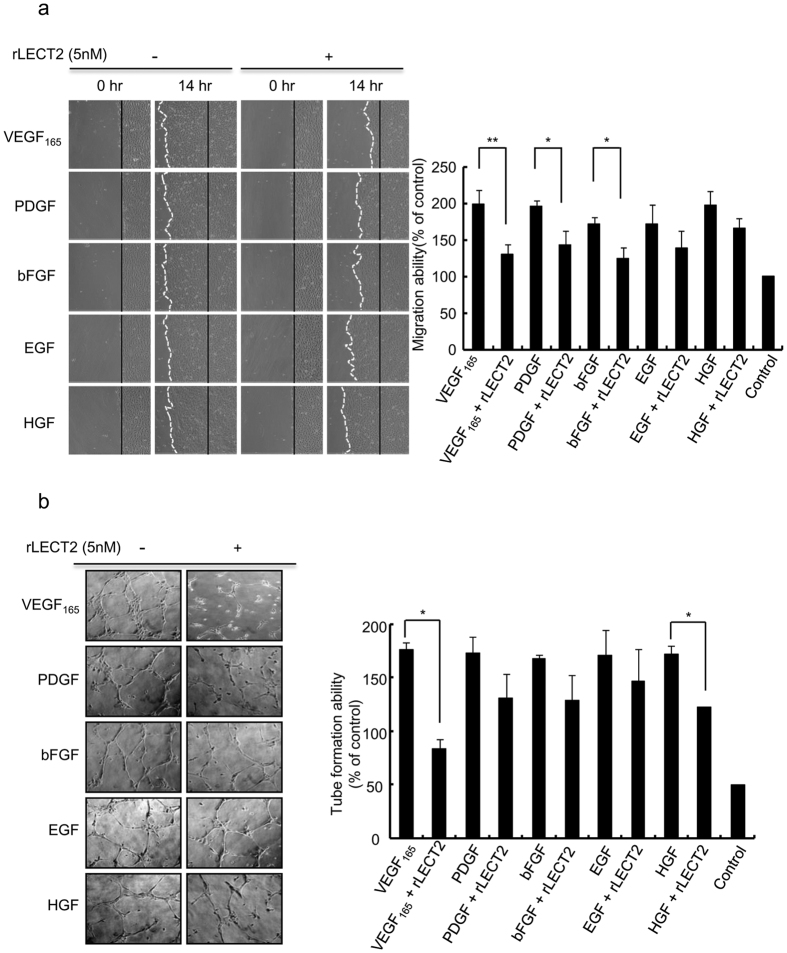
rLECT2 protein inhibits migration and tube formation induced by angiogenic factors in HUVECs. (**a**) HUVECs were seeded in a 24-well plate, treated with VEGF_165_ (50 ng/mL), PDGF (50 ng/mL), bFGF (50 ng/mL), EGF (50 ng/mL), or HGF (40 ng/mL) alone or combined with rLECT2 protein (5 nM) as indicated for 14 h, and subjected to Migration assay. (**b**) HUVECs were seeded onto a Matrigel layer in a 24-well plate, treated with different angiogenic factors as indicated for 6 h, and subjected to tube formation assay. The experiments were repeated three times with similar results. Data are presented as the mean ± SD in triplicate. A paired Student *t*-test was used to evaluate the differences in the migration ratio between groups treated with angiogenic factors alone and in combination with rLECT2 protein. *P < 0.05; **P < 0.01.

To determine whether LECT2 protein interferes with specific angiogenic factors, we first purified rLECT2 protein and performed migration and tube formation assays with HUVECs. The addition of VEGF_165_ (50 ng/mL), PDGF (50 ng/mL), bFGF (30 ng/mL), epidermal growth factor (EGF; 50 ng/mL), and hepatocyte growth factor (HGF; 40 ng/mL) to starvation medium significantly induced HUVEC migration and tube formation. In contrast, the addition of rLECT2 (5 nM) to HUVECs treated with angiogenic factors inhibited VEGF_165_-, PDGF-, and bFGF-induced HUVEC migration by 34%, 27%, and 27%, respectively, and HGF- and VEGF_165_-induced tube formation by 30% and 52%, respectively (Fig. 3a,b). We also used a human phospho-RTK array to detect alterations in phosphorylated RTKs in HUVECs after LECT2-based treatment. We found that VEGFR2 phosphorylation was strongly inhibited by treatment with rLECT2 protein (Supplementary Fig. S1). These data suggested that rLECT2 protein inhibits tumor angiogenesis by inhibiting the activity of specific angiogenic factors and receptors, particularly the VEGF_165_/VEGFR2 axis.

### rLECT2 protein suppresses VEGF_165_-induced angiogenesis in HUVECs

**Figure 4:**
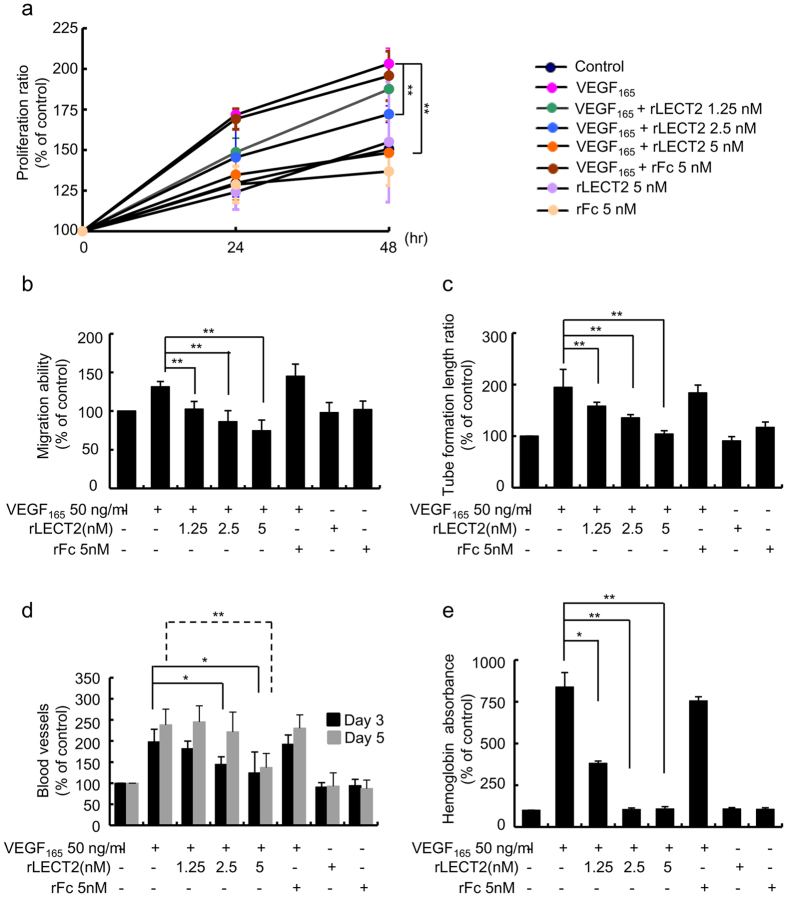
rLECT2 protein suppresses VEGF-induced angiogenic responses. (**a**) Proliferation ratios for HUVECs seeded in a 96-well plate and treated with VEGF_165_ (50 ng/mL) alone or combined with various concentrations of rLECT2 protein (1.25, 2.50, and 5.00 nM) as indicated for 24 and 48 h. Cell growth was measured using an MTT assay. (**b**) A confluent HUVEC monolayer was wounded with a blue pipette tip and then exposed to fresh M199 medium (control) or a medium containing VEGF_165_ (50 ng/mL) with various concentrations of rLECT2 protein (0–5 nM) for 14 h. The width of the wound on the monolayer was measured to determine migration ability of HUVECs. Images of migration HUVECs were obtained and analyzed using the Image-Pro Plus software program (version 4.5). (**c**) HUVECs were seeded onto a Matrigel layer in a 24-well plate and treated with VEGF_165_ (50 ng/mL) combined with various concentrations of rLECT2 protein as indicated for 6 h. Tube formation was determined by manual counting of the tubular structures in low-power fields (40×). (**d**) CAM blood vessel formation. CAMs of 9-day-old chicken embryos were incubated with VEGF_165_ alone (50 ng/mL) or combined with various concentrations of rLECT2 protein as indicated for 1–4 days and then photographed. (**e**) A Matrigel mixture containing VEGF alone or combined with various concentrations of rLECT2 protein as indicated was injected subcutaneously into nude mice at sites lateral to the abdominal midline. Matrigel plugs were recovered from the mice and photographed immediately 10 days later. The hemoglobin absorbance was measured to determine hemoglobin levels in the plugs. The data are presented as the mean ± SD. Each treatment was performed in triplicate, and the assays were repeated at least three times. *P ＜ 0.05; **P ＜ 0.01.

VEGF expression levels are highly correlated with the disease progression and clinical outcome of HCC^21^,^22^. Thus, we asked whether LECT2 could inhibit VEGF_165_-induced angiogenic response in HUVECs. To do so, we first investigated the effects of LECT2 treatment on the proliferation, migration, and tube formation of VEGF_165_-treated HUVECs (Fig. 4a–c). HUVECs were treated with various concentrations of rLECT2 protein with or without VEGF_165_ (50 ng/mL) for 24 and 48 h. As expected, VEGF_165_-induced proliferation of HUVECs decreased significantly in the presence of 2.5 nM and 5 nM rLECT2 protein (*p* < 0.01) (Fig. 4a), indicating rLECT2 was effective in inhibiting VEGF_165_-induced proliferation of HUVECs. In addition, rLECT2 protein suppressed VEGF_165_-induced HUVEC migration (Fig. 4b; Supplementary Fig. S2a) and tube formation (Fig. 4c; Supplementary Fig. S2b) in a dose-dependent manner.

Next, we examined whether rLECT2 protein inhibits VEGF_165_-induced angiogenesis using an *ex vivo* CAM assay and *in vivo* Matrigel plug assay. The results indicated that VEGF_165_ increased the capillary bed area of the chorioallantois in the CAMs more so than did the non-treatment control and that rLECT2 protein suppressed the VEGF_165_-induced capillary bed area in a dose-dependent manner (Fig. 4d; Supplementary Fig. S2c). Moreover, we implanted Matrigel plugs containing control phosphate-buffered saline (PBS) or VEGF_165_ with or without rLECT2 protein into C57BL/6J mice and recovered the plugs 10 days later. Macroscopic analysis demonstrated that the plugs containing VEGF_165_ and rLECT2 protein were much paler than those containing only VEGF_165_ (Supplementary Fig. S2d), indicating the loss of hemoglobin. Similarly, the hemoglobin level was markedly higher in plugs containing VEGF_165_ than in those containing PBS only. Hemoglobin induction by VEGF_165_ was largely inhibited in plugs containing both VEGF_165_ and rLECT2 protein (2.5 nM and 5.0 nM) (Fig. 4e).

Vascular permeability is a prominent early feature of pathological angiogenesis and highly dependent on VEGF activation. Therefore, we investigated whether rLECT2 protein can target VEGF_165_-induced vascular permeability. The results demonstrated that rLECT2 protein suppressed vascular permeability in a dose-dependent manner (Supplementary Fig. S3a). Moreover, treatment with rLECT2 protein blocked permeable dye out of the tumor vessels more so than in the VEGF_165_ group as demonstrated by the *ex vivo* Miles assay (Supplementary Fig. S3b). Taken together, these findings strongly suggested that the rLECT2 protein attenuates VEGF_165_-induced angiogenic effects *in vitro*, *ex vivo*, and *in vivo*.

### rLECT2 downregulates VEGF_165_-induced VEGFR2 tyrosine phosphorylation and downstream protein signaling

**Figure 5:**
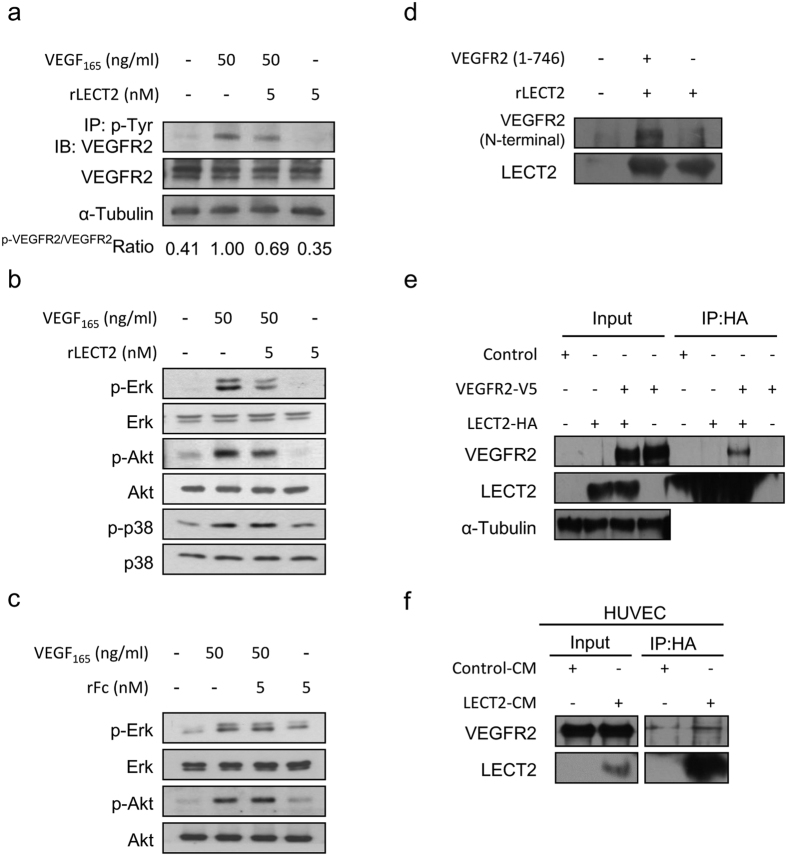
Effects of treatment with rLECT2 on VEGF_165_-stimulated VEGFR2 tyrosine phosphorylation and downstream protein expression in HUVECs. (**a**) Immunoblot of phosphorylation VEGFR in HUVECs. Serum-starved HUVECs were incubated with indicated treatment for 15 min. Cell extracts were subjected to immunoprecipitation (IP) with an antibody against the phosphotyrosine pY99. Precipitated proteins were analyzed via immunoblotting (IB) with an antibody against VEGFR2 (KDR) present in pY-VEGFR2. The same blots were subsequently reprobed with antibodies against VEGFR2 present in the receptor. (**b**) Immunoblot showing expression of the indicate proteins in HUVECs. HUVECs were serum-starved for 8 h and then treated with rLECT2 protein (5 nM) for 15 min prior to treatment with VEGF. (**c**) HUVECs were serum-starved for 8 h and then treated with rFc-Tag protein (5 nM) as negative control for 15 min prior to treatment with VEGF. Each treatment was performed in triplicate. (**d**) An *in vitro* binding assay to detect LECT2 and VEGFR2 binding. An Fc-tagged rLECT2 protein and His-tagged VEGFR2 extracellular domain (1–746 amino acids) were incubated and purified using a nickel-affinity column. The washed precipitates were then subjected to the western blot. (**e**) 293T cells were transfected with a control vector, HA-tagged LECT2 (LECT2-HA), or V5-tagged VEGFR2 (VEGFR2-V5) as indicated. Cell lysates were immunoprecipitated with an HA antibody and then subjected to immunoblotting with the indicated antibodies. (**f**) Endogenous interactions between LECT2 and VEGFR2 in HUVECs were evaluated. The HUVECs were treated with 293T cell-expressing control or LECT2 CM for 30 min, and cell lysates were harvested. HUVEC lysates were immunoprecipitated with an antibody as indicated.

To delineate the molecular mechanisms underlying rLECT2-inhibited VEGF-induced angiogenesis, we first examined VEGFR2 and its tyrosine kinase phosphorylation status in HUVECs. Consistent with results from our phospho-RTK array screening described above, we found that phosphorylation of VEGFR2 was markedly reduced after rLECT2-based treatment (Fig. 5a). VEGFR2 undergoes dimerization in cells and subsequently induces the activation of intracellular pathways, including Src, phosphoinositide 3-kinase/AKT, and Raf/mitogen-activated protein kinase kinase/extracellular signal-regulated kinase (ERK)^6^,^23^,^24^,^25^,^26^,^27^. We found that phosphorylation of ERK and AKT protein induced by VEGF_165_ stimulation decreased under rLECT2-based treatment, whereas phosphorylation of p38 was not affected (Fig. 5b). In comparison, control rFc protein had no effect on VEGF-induced signaling in HUVECs (Fig. 5c). Because we previously found that LECT2 bound directly to MET and suppress its phosphorylation^17^, we next performed an *in vitro* binding assay to determine whether LECT2 also inhibits VEGFR2 phosphorylation by binding to VEGFR2. Our data revealed that rLECT2 protein binds directly to the extracellular domain (1–746 amino acids) of recombinant VEGFR2 protein (Fig. 5d). Co-immunoprecipitation experiments of 293T human embryonic kidney cells co-transfected with LECT2 and VEGFR2 also demonstrated the interaction between LECT2 and VEGFR2 in (Fig. 5e) as well as in HUVECs treated with CM from 293T cells overexpressing LECT2 (Fig. 5f). These results suggested that LECT2 protein inhibits VEGF_165_-induced VEGFR2 phosphorylation and downstream signaling via direct binding with VEGFR2.

### LECT2 expression is negatively correlated with angiogenesis in HCC patients

**Figure 6:**
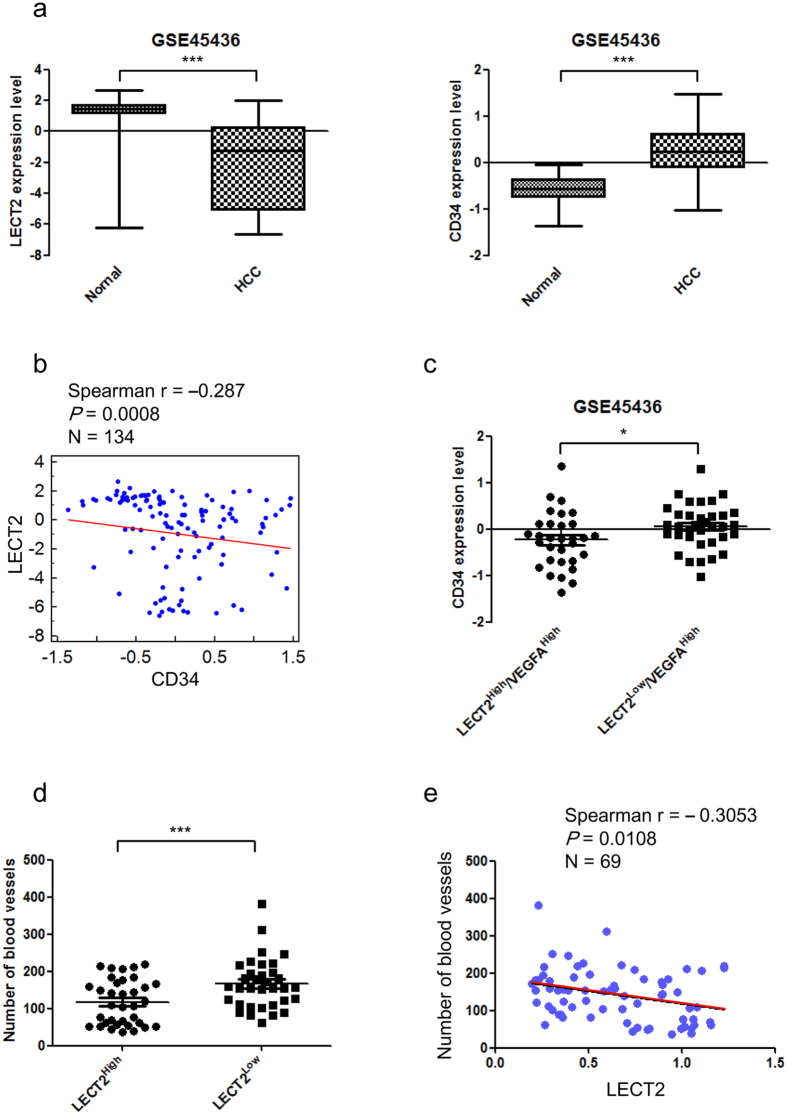
LECT2 expression is inversely correlated with angiogenesis in HCC patients. (**a–e**) Analysis of the correlation between LECT2 and angiogenic marker (CD34) expression in HCC patients using data from the Gene Expression Omnibus database (GSE45436). (A left) Comparison of the LECT2 gene expression levels in normal liver tissue and HCC samples. (A right) Comparison of the CD34 gene expression levels in normal liver tissue and HCC samples. (**b**) Gene expression scatter diagrams for LECT2 versus CD34. The blue dots represent the expression levels in individual samples in the cohort, and a regression line is shown. (**c**) Correlation between CD34 and LECT2 expression with high VEGF_165_ gene expression. (**d**) Correlation between LECT2 protein expression and MVD in HCC patients. The LECT2 protein expression levels in 73 HCC samples were determined via immunoblotting. MVD was analyzed by staining tissue sections immunohistochemically and then evaluating three highly vascularized areas per tumor at high magnification (200×). The total number of microvessels was determined for each area, and the average number was recorded for each tumor. (**e**) Protein expression scatter diagrams for LECT2 versus MVD from HCC patients.

To determine the clinical significance of LECT2 expression for HCC patients in our study, we used the Gene Expression Omnibus (GSE45436) and The Cancer Genome Atlas databases to analyzed the LECT2 and angiogenesis biomarker gene expression correlation (CD34) in HCC patients (Fig. 6a–e); Supplementary Fig. S3). As expected, LECT2 gene expression was markedly lower in HCCs than in normal liver tissue samples (Fig. 6a, left). Consistent with the highly angiogenic nature of HCC, CD34 gene expression was higher in HCCs than in normal tissue (Fig. 6a, right). We also examined the correlation between LECT2 and CD34 expression in HCC patients. The data demonstrated that LECT2 expression was inversely correlated with CD34 expression (n = 134; *P* = 0.0008) (Fig. 6b; Supplementary Fig. S4a). Of note, samples with high LECT2 expression tended to have low CD34 expression, even in the presence of high VEGF_165_ expression (Fig. 6c; Supplementary Fig. S4b and S4c). Furthermore, we quantified the microvascular density (MVD) of HCC patient liver tissues by immunohistochemical staining for pan-endothelial cell antigen. LECT2 expression and MVD were inversely correlated (n = 69; *P* = 0.0108; Fig. 6d,e). These data indicated that LECT2 expression was inversely associated with HCC angiogenesis.

## Discussion

Liver tumors have marked vascular abnormalities, which leads to hypoxia and contributes to tumor progression. During tumor angiogenesis, expression of proangiogenic factors in tumor cells exceeds the release of antiangiogenic molecules. In this study, we found that treatment with LECT2 inhibited tumor growth but not cancer cell proliferation in a xenograft mice model of HCC. Moreover, we showed that LECT2 markedly inhibited VEGF_165_-induced angiogenic activities, including proliferation, migration, tube formation, and vascular permeability, in HUVECs. Importantly, LECT2 inhibition of angiogenesis may result from direct binding of LECT2 to VEGFR and downregulation of VEGFR2-mediated ERK and AKT activation. In HCC patient samples, LECT2 expression was negatively correlated with angiogenesis marker expression.

The VEGF/VEGFR axis is recognized as an important regulator of tumor angiogenesis in HCC^28^,^29^. Also, inhibition of angiogenesis is a potential therapeutic for HCC. Previous reports demonstrated that LECT1, also known as chondromodulin-I, is an endogenous angiogenesis inhibitor in cartilage, cardiac valvular, connective tissue, retinal endothelial, and vascular endothelial cells^30^,^31^,^32^,^33^,^34^,^35^,^36^,^37^. Miura *et al*.^30^ showed that LECT1 impaired VEGF_165_-stimulated migration of vascular endothelial cells by destabilizing lamellipodial extensions and that LECT1 markedly reduced VEGF_165_-induced Rac1 activity in HUVEC. However, the sequences and structures of LECT1 and LECT2 are not similar^9^. LECT2 is a tumor suppressor in HCCs^16^ but it is not yet clear whether LECT2 also regulates the angiogenic activity in specific tissues. Here, we demonstrated for the first time that treatment with rLECT2 protein inhibits angiogenic activities induced by multiple angiogenic factors, particularly VEGF_165_. Two of the major signaling pathways stimulated by VEGF_165_ are the Raf-1/mitogen-activated protein kinase kinase/ERK cascade and phosphoinositide 3-kinase/AKT^38^. Our data demonstrated that rLECT2 protein suppressed ERK and AKT activation in HUVEC after VEGFR2 stimulation. Furthermore, our *in vitro* binding assay and co-immunoprecipitation data demonstrated that LECT2 binds directly to VEGFR2. In addition, ectopic expression of LECT2 in our xenograft model of HCC reduced MVD. In patient samples, expression of LECT2 was negatively correlated with that of angiogenesis markers CD34 and MVD. All of these findings suggest that LECT2 is a novel antiangiogenic factor and suppresses VEGF_165_-induced angiogenesis and tumor growth in HCC patients.

Although a previous study by our group indicated that LECT2 expression suppressed HCC vascular invasion and metastasis by blocking HGF/MET signaling^17^, the role of LECT2 in liver tumor microenvironments is not well understood. Many studies have demonstrated that LECT2 regulates inflammation and immunomodulation. For example, treatment with LECT2 induced macrophage activation in a mouse model of bacterial sepsis^39^. LECT2 also negatively regulates the homeostasis of natural killer T cells in the liver^15^. Recently, Hwang *et al*.^40^ showed that LECT2 induced an atherosclerotic inflammatory reaction via CD209-mediated c-Jun N-terminal kinase phosphorylation in human endothelial cells and that LECT2 induces the expression of proinflammatory cytokines, such as tumor necrosis factor-α, monocyte chemotactic protein 1, and IL-1β. In the present study, we further demonstrated that LECT2 suppressed tumor angiogenesis, inhibiting tumor growth in immunodeficient HCC mouse model. In addition to tumor angiogenesis in HCC, we also found that LECT2 reduced MVD and tumor growth in ectopic expression of LECT2 in B16F1 mouse melanoma model (data not shown), suggesting LECT2 broadly suppressed tumorigenesis via tumor angiogenesis. As tumor angiogenesis and inflammation are key events in tumor progression^41^, these studies suggested that LECT2 plays an important role in regulation of homeostasis of the tumor microenvironment.

On the basis of our findings, LECT2 is a potential therapeutic agent for HCC because it inhibits both tumor angiogenesis (anti-VEGFR2) and metastasis (anti-MET). VEGF/VEGFR and HGF/MET are important signaling pathways in promotion of HCC progression. Many inhibitors target these two pathways. Currently, sorafenib is the only US. Food and Drug Administration-approved VEGFR-targeting treatment of unresectable HCC. However, recent studies demonstrated that antiangiogenic treatment may accelerate local invasion and distant metastasis^42^,^43^. In addition, MET expression is upregulated in tumor cells after treatment with sorafenib, resulting in hepatocellular tumor metastasis^44^,^45^. Our previous study indicated that LECT2 is a MET antagonist that suppresses vascular invasion in HCCs^17^. Our current study further suggested that LECT2 binds to VEGFR2 and inhibit HCC angiogenesis. Therefore, LECT2 could inhibit both tumor growth and metastasis by simultaneously targeting MET and VEGFR2 in HCC patients.

Overall, we revealed a significant correlation between LECT2 expression and tumor angiogenesis in HCC progression. Our findings support the future development of LECT2-based therapies targeting stromal signaling and solid tumor cells that depend on VEGF signaling.

## Additional Information

**How to cite this article**: Chen, C.-K. *et al*. Inhibition of VEGF_165_/VEGFR2-dependent signaling by LECT2 suppresses hepatocellular carcinoma angiogenesis. *Sci. Rep*. **6**, 31398; doi: 10.1038/srep31398 (2016).

## Supplementary Material

Supplementary Information

## References

[b1] Zhu A. X., Duda D. G., Sahani D. V. & Jain R. K. HCC and angiogenesis: possible targets and future directions. Nature reviews. Clinical oncology 8, 292–301, doi: 10.1038/nrclinonc.2011.30 (2011). PMC3266719 21386818

[b2] Bupathi M., Kaseb A., Meric-Bernstam F. & Naing A. Hepatocellular carcinoma: Where there is unmet need. Molecular oncology 9, 9, doi: 10.1016/j.molonc.2015.06.005 (2015). PMC5528787 26160430

[b3] Muto J., Shirabe K., Sugimachi K. & Maehara Y. Review of angiogenesis in hepatocellular carcinoma. Hepatology research: the official journal of the Japan Society of Hepatology 45, 1–9, doi: 10.1111/hepr.12310 (2015). 24533487

[b4] Carmeliet P. & Jain R. K. Molecular mechanisms and clinical applications of angiogenesis. Nature 473, 298–307, doi: 10.1038/nature10144 (2011). 21593862 PMC4049445

[b5] Scartozzi M. . VEGF and VEGFR genotyping in the prediction of clinical outcome for HCC patients receiving sorafenib: the ALICE-1 study. International journal of cancer. Journal international du cancer 135, 1247–1256, doi: 10.1002/ijc.28772 (2014). 24510746

[b6] Matsumoto T. & Claesson-Welsh L. VEGF receptor signal transduction. Sci STKE 2001, RE21 (2001). 11741095 10.1126/stke.2001.112.re21

[b7] Neufeld G., Cohen T., Gengrinovitch S. & Poltorak Z. Vascular endothelial growth factor (VEGF) and its receptors. Faseb J 13, 9–22 (1999). 9872925

[b8] Suzuki F. Roles of cartilage matrix proteins, chondromodulin-I and -II, in endochondral bone formation: a review. Connective tissue research 35, 303–307 (1996). 9084668 10.3109/03008209609029204

[b9] Shukunami C. . Molecular cloning of mouse and bovine chondromodulin-II cDNAs and the growth-promoting actions of bovine recombinant protein. Journal of biochemistry 125, 436–442 (1999). 10050029 10.1093/oxfordjournals.jbchem.a022305

[b10] Yamagoe S., Mizuno S. & Suzuki K. Molecular cloning of human and bovine LECT2 having a neutrophil chemotactic activity and its specific expression in the liver. Biochimica et biophysica acta 1396, 105–113 (1998). 9524238 10.1016/s0167-4781(97)00181-4

[b11] Ito M. . Expression, oxidative refolding, and characterization of six-histidine-tagged recombinant human LECT2, a 16-kDa chemotactic protein with three disulfide bonds. Protein expression and purification 27, 272–278 (2003). 12597887 10.1016/s1046-5928(02)00634-4

[b12] Yamagoe S., Kameoka Y., Hashimoto K., Mizuno S. & Suzuki K. Molecular cloning, structural characterization, and chromosomal mapping of the human LECT2 gene. Genomics 48, 324–329, doi: 10.1006/geno.1997.5198 (1998). 9545637

[b13] Ohtomi M., Nagai H., Ohtake H., Uchida T. & Suzuki K. Dynamic change in expression of LECT2 during liver regeneration after partial hepatectomy in mice. Biomedical research 28, 247–253 (2007). 18000337 10.2220/biomedres.28.247

[b14] Sato Y. . Serum LECT2 level as a prognostic indicator in acute liver failure. Transplantation proceedings 36, 2359–2361, doi: 10.1016/j.transproceed.2004.07.007 (2004). 15561249

[b15] Saito T. . Increase in hepatic NKT cells in leukocyte cell-derived chemotaxin 2-deficient mice contributes to severe concanavalin A-induced hepatitis. Journal of immunology 173, 579–585 (2004). 10.4049/jimmunol.173.1.579 15210819

[b16] Ong H. T. . The tumor suppressor function of LECT2 in human hepatocellular carcinoma makes it a potential therapeutic target. Cancer gene therapy 18, 399–406, doi: 10.1038/cgt.2011.5 (2011). 21394108

[b17] Chen C. K. . Leukocyte cell-derived chemotaxin 2 antagonizes MET receptor activation to suppress hepatocellular carcinoma vascular invasion by protein tyrosine phosphatase 1B recruitment. Hepatology 59, 974–985, doi: 10.1002/hep.26738 (2014). 24114941

[b18] Gimbrone M. A. Jr., Cotran R. S. & Folkman J. Human vascular endothelial cells in culture. Growth and DNA synthesis. J Cell Biol 60, 673–684 (1974). 4363161 10.1083/jcb.60.3.673 PMC2109230

[b19] Chen H.-L., Chiu T.-S., Chen P.-J. & Chen D.-S. Cytogenetic studies on human liver cancer cell lines. Cancer Genetics and Cytogenetics 65, 161–166, doi: 10.1016/0165-4608(93)90227-D (1993). 8384076

[b20] Xiao D. & Singh S. V. Phenethyl isothiocyanate inhibits angiogenesis *in vitro* and *ex vivo*. Cancer Res 67, 2239–2246 (2007). 17332354 10.1158/0008-5472.CAN-06-3645

[b21] Schoenleber S. J., Kurtz D. M., Talwalkar J. A., Roberts L. R. & Gores G. J. Prognostic role of vascular endothelial growth factor in hepatocellular carcinoma: systematic review and meta-analysis. British journal of cancer 100, 1385–1392, doi: 10.1038/sj.bjc.6605017 (2009). 19401698 PMC2694418

[b22] Poon R. T. . High serum vascular endothelial growth factor levels predict poor prognosis after radiofrequency ablation of hepatocellular carcinoma: importance of tumor biomarker in ablative therapies. Annals of surgical oncology 14, 1835–1845, doi: 10.1245/s10434-007-9366-z (2007). 17406950

[b23] Dayanir V., Meyer R. D., Lashkari K. & Rahimi N. Identification of tyrosine residues in vascular endothelial growth factor receptor-2/FLK-1 involved in activation of phosphatidylinositol 3-kinase and cell proliferation. The Journal of biological chemistry 276, 17686–17692, doi: 10.1074/jbc.M009128200 (2001). 11278468

[b24] Jiang B. H. & Liu L. Z. PI3K/PTEN signaling in angiogenesis and tumorigenesis. Advances in cancer research 102, 19–65, doi: 10.1016/S0065-230X(09)02002-8 (2009). 19595306 PMC2933405

[b25] Sun Z. . VEGFR2 induces c-Src signaling and vascular permeability *in vivo* via the adaptor protein TSAd. The Journal of experimental medicine 209, 1363–1377, doi: 10.1084/jem.20111343 (2012). 22689825 PMC3405501

[b26] Claesson-Welsh L. & Welsh M. VEGFA and tumour angiogenesis. Journal of internal medicine 273, 114–127, doi: 10.1111/joim.12019 (2013). 23216836

[b27] Mavria G. . ERK-MAPK signaling opposes Rho-kinase to promote endothelial cell survival and sprouting during angiogenesis. Cancer cell 9, 33–44, doi: 10.1016/j.ccr.2005.12.021 (2006). 16413470

[b28] Olsson A. K., Dimberg A., Kreuger J. & Claesson-Welsh L. VEGF receptor signalling - in control of vascular function. Nat Rev Mol Cell Biol 7, 359–371 (2006). 16633338 10.1038/nrm1911

[b29] Ferrara N., Gerber H. P. & LeCouter J. The biology of VEGF and its receptors. Nature medicine 9, 669–676, doi: 10.1038/nm0603-669 (2003). 12778165

[b30] Miura S. . Impairment of VEGF-A-stimulated lamellipodial extensions and motility of vascular endothelial cells by chondromodulin-I, a cartilage-derived angiogenesis inhibitor. Experimental cell research 316, 775–788, doi: 10.1016/j.yexcr.2009.12.009 (2010). 20026108

[b31] Shukunami C. & Hiraki Y. Chondromodulin-I and tenomodulin: the negative control of angiogenesis in connective tissue. Current pharmaceutical design 13, 2101–2112 (2007). 17627542 10.2174/138161207781039751

[b32] Yoshioka M. . Chondromodulin-I maintains cardiac valvular function by preventing angiogenesis. Nature medicine 12, 1151–1159, doi: 10.1038/nm1476 (2006). 16980969

[b33] Hiraki Y. & Shukunami C. Angiogenesis inhibitors localized in hypovascular mesenchymal tissues: chondromodulin-I and tenomodulin. Connective tissue research 46, 3–11, doi: 10.1080/03008200590935547 (2005). 16019413

[b34] Oshima Y. . Expression and localization of tenomodulin, a transmembrane type chondromodulin-I-related angiogenesis inhibitor, in mouse eyes. Investigative ophthalmology & visual science 44, 1814–1823 (2003). 12714610 10.1167/iovs.02-0664

[b35] Hayami T. . Expression of the cartilage derived anti-angiogenic factor chondromodulin-I decreases in the early stage of experimental osteoarthritis. The Journal of rheumatology 30, 2207–2217 (2003). 14528519

[b36] Funaki H. . Expression and localization of angiogenic inhibitory factor, chondromodulin-I, in adult rat eye. Investigative ophthalmology & visual science 42, 1193–1200 (2001). 11328727

[b37] Hayami T. . Specific loss of chondromodulin-I gene expression in chondrosarcoma and the suppression of tumor angiogenesis and growth by its recombinant protein *in vivo*. FEBS letters 458, 436–440 (1999). 10570955 10.1016/s0014-5793(99)01201-6

[b38] Karkkainen M. J. & Petrova T. V. Vascular endothelial growth factor receptors in the regulation of angiogenesis and lymphangiogenesis. Oncogene 19, 5598–5605, doi: 10.1038/sj.onc.1203855 (2000). 11114740

[b39] Lu X. J. . LECT2 protects mice against bacterial sepsis by activating macrophages via the CD209a receptor. The Journal of experimental medicine 210, 5–13, doi: 10.1084/jem.20121466 (2013). 23254286 PMC3549712

[b40] Hwang H. J. . LECT2 induces atherosclerotic inflammatory reaction via CD209 receptor-mediated JNK phosphorylation in human endothelial cells. Metabolism: clinical and experimental 64, 1175–1182, doi: 10.1016/j.metabol.2015.06.001 (2015). 26123523

[b41] Albini A. & Sporn M. B. The tumour microenvironment as a target for chemoprevention. Nature reviews. Cancer 7, 139–147, doi: 10.1038/nrc2067 (2007). 17218951

[b42] Paez-Ribes M. . Antiangiogenic therapy elicits malignant progression of tumors to increased local invasion and distant metastasis. Cancer cell 15, 220–231, doi: 10.1016/j.ccr.2009.01.027 (2009). 19249680 PMC2874829

[b43] Ebos J. M. . Accelerated metastasis after short-term treatment with a potent inhibitor of tumor angiogenesis. Cancer cell 15, 232–239, doi: 10.1016/j.ccr.2009.01.021 (2009). 19249681 PMC4540346

[b44] Sennino B. . Suppression of tumor invasion and metastasis by concurrent inhibition of c-Met and VEGF signaling in pancreatic neuroendocrine tumors. Cancer discovery 2, 270–287, doi: 10.1158/2159-8290.CD-11-0240 (2012). 22585997 PMC3354652

[b45] Zhang W. . Sorafenib down-regulates expression of HTATIP2 to promote invasiveness and metastasis of orthotopic hepatocellular carcinoma tumors in mice. Gastroenterology 143, 1641–1649, e1645, doi: 10.1053/j.gastro.2012.08.032 (2012). 22922424

