# Supplementary material for: Inhibition of VEGF165/VEGFR2-dependent signaling by LECT2 suppresses hepatocellular carcinoma angiogenesis
Source: Sci Rep. 2016 Aug 10;6:31398. doi: 10.1038/srep31398 (PMC4979047; doi:10.1038/srep31398)
Supplement: Supplementary Information [file srep31398-s1.doc]

**Supplementary Information**

**Inhibition of VEGF_165_/VEGFR2-dependent signaling by LECT2 suppresses hepatocellular carcinoma angiogenesis**

Chi-Kuan Chen,^1,2^,* Wen-Hsuan Yu,^3,4,5^,* Tsu-Yao Cheng,^6,7^ Min-Wei Chen,^8^ Chia-Yi Su,^1^ Yi-Chieh Yang,^9^ Tsang-Chih Kuo,^10^ Ming-Tsan Lin,^11,12^ Ya-Chi Huang^13^, Michael Hsiao,^1^ Kuo-Tai Hua,^2,†^ Mien-Chie Hung,^3,4,5,†^ Min-Liang Kuo^2,10,†^

^1^Genomics Research Center, Academia Sinica, Taipei, Taiwan

^2^Graduate Institute of Toxicology, College of Medicine, National Taiwan University, Taipei, Taiwan

^3^Department of Molecular and Cellular Oncology, The University of Texas MD Anderson Cancer Center, Houston, TX, USA

^4^The University of Texas Graduate School of Biomedical Sciences at Houston, Houston TX, USA

^5^Center for Molecular Medicine and Graduate Institute of Cancer Biology, China Medical University, Taichung, Taiwan

^6^Department of Laboratory Medicine, National Taiwan University Hospital, Taipei, Taiwan

^7^Department of Internal Medicine, National Taiwan University Hospital, Taipei, Taiwan

^8^Department of Oncology, National Taiwan University Hospital, Taipei, Taiwan

^9^ Graduate Institute of Oncology, College of Medicine, National Taiwan University, Taipei, Taiwan.

^10^Institute of Biomedical Sciences, College of Life Science, National Taiwan University, Taipei, Taiwan

^11^Department of Surgery, National Taiwan University Hospital, and National Taiwan University College of Medicine, Taipei, Taiwan

^12^Department of Primary Care Medicine, National Taiwan University Hospital and National Taiwan University College of Medicine, Taipei, Taiwan

^13^Graduate Institute of Microbiology, College of Medicine, National Taiwan University, Taipei, Taiwan

*These authors contributed equally to this work.

**Correspondence should be addressed to** Kuo-Tai Hua, National Taiwan University College of Medicine, No. 1, Jen-Ai Road, Taipei 100, Taiwan. Tel.: 886-2-23123456 ext. 88615; fax: 886-2-23958341; E-mail address: kthua@ntu.edu.tw; Mien-Chie Hung, Department of Molecular and Cellular Oncology, Unit 108, The University of Texas MD Anderson Cancer Center, 1515 Holcombe Boulevard, Houston, Texas 77030, USA. Tel.: 713-792-3668; fax: 713-794-3270; E-mail address: mhung@mdanderson.org; or Min-Liang Kuo, National Taiwan University College of Medicine, No. 1, Jen-Ai Road, Taipei 100, Taiwan. Tel.: 886-2-23123456 ext. 88600; fax: 886-2-23410217; E-mail address: kuominliang@ntu.edu.tw.

**
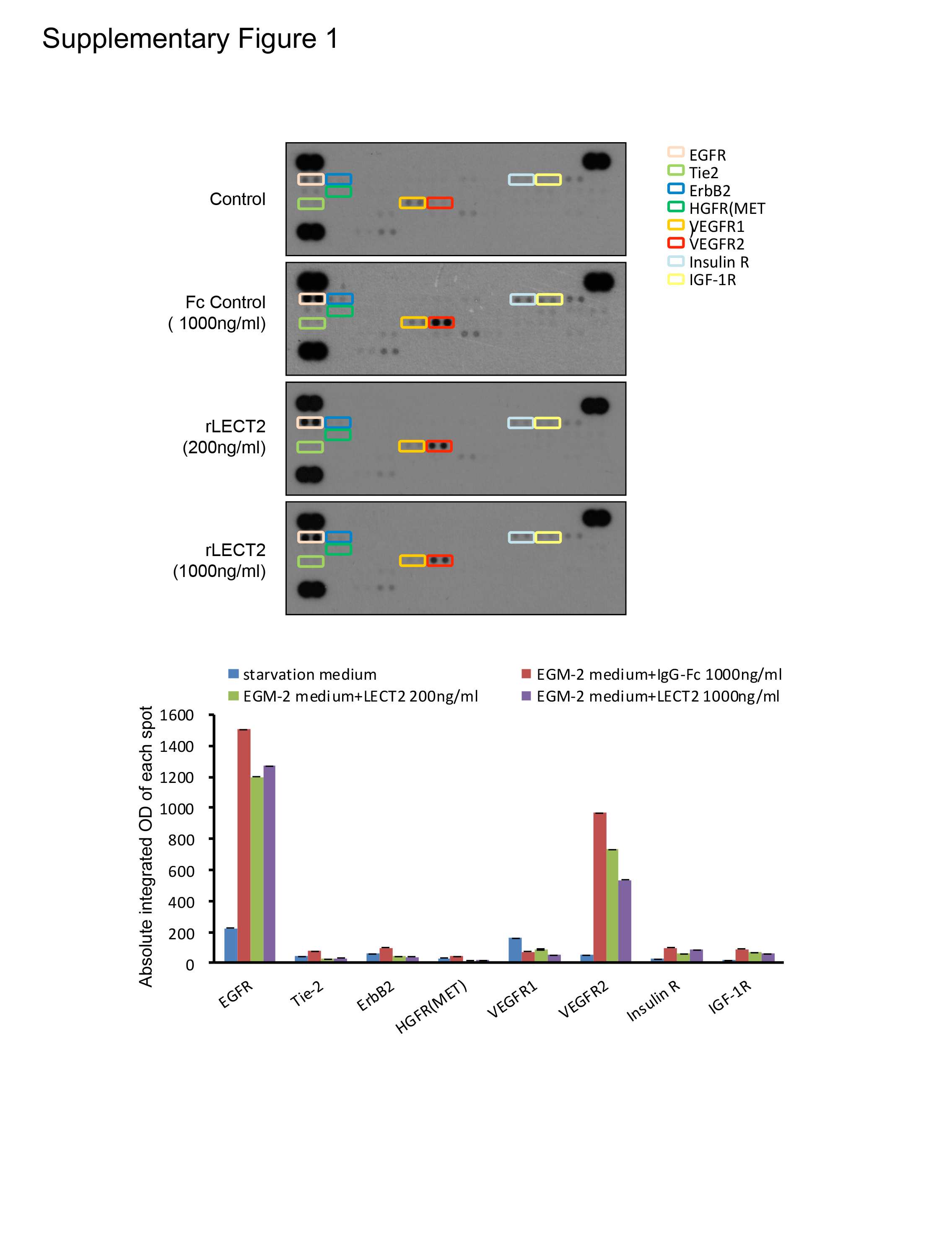
Supplementary Fig. S1. Analysis of the effects of rLECT2 on RTKs via phospho-RTK arrays.** HUVECs treated with rFc-tagged LECT2 (200 and 1000 ng/ml) and Fc (1000 ng/ml) protein were subjected to a phospho-RTK array. Upper panel: equal amounts of total protein extracts were incubated with the arrays. The spots (colored rectangles) indicate activated RTKs. Lower panel: quantification of activated RTKs.

**
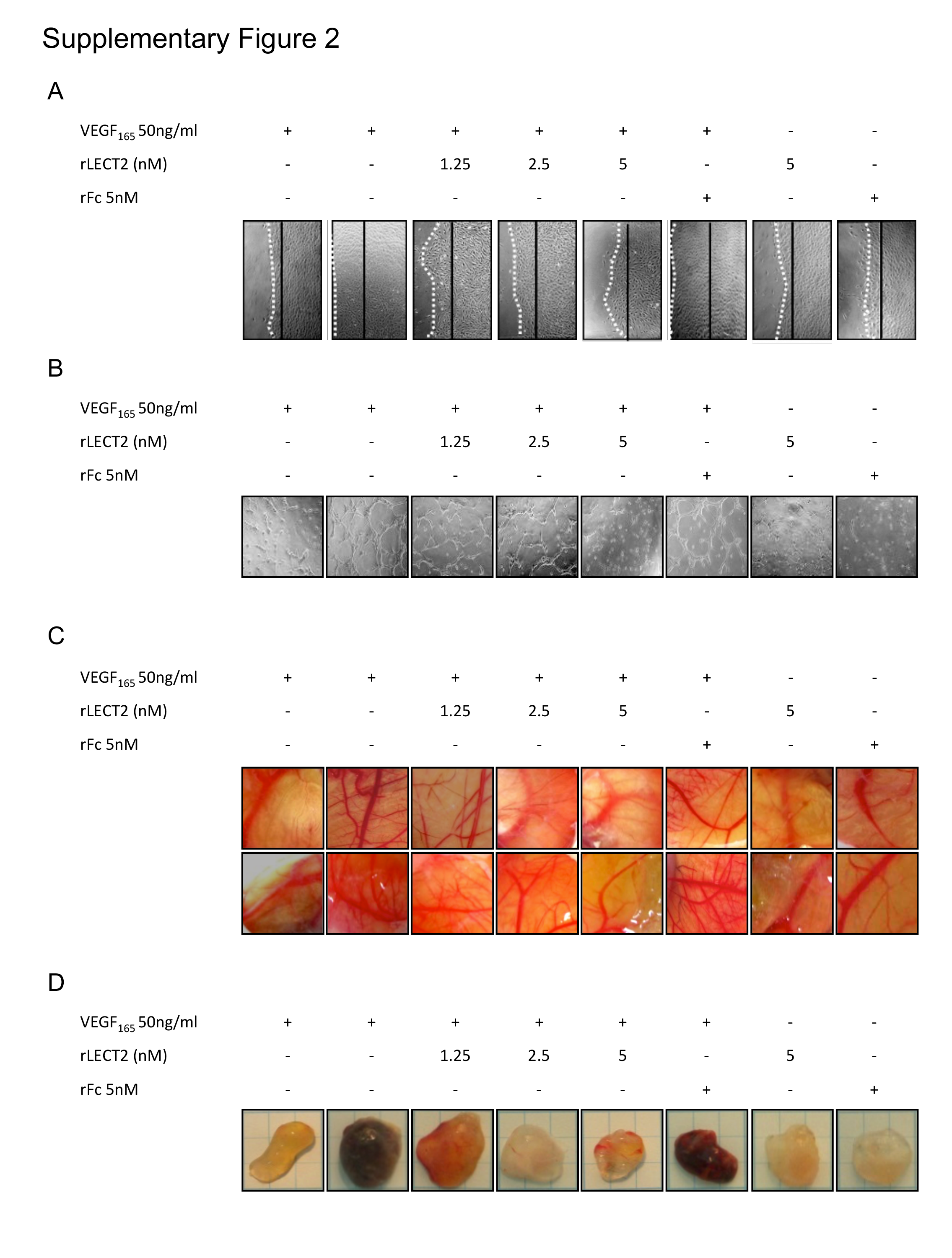
Supplementary Fig. S2. rLECT2 protein suppresses VEGF-induced angiogenic responses.** (A) Effect of rLECT2 protein expression on VEGF-induced HUVEC migration. The images were obtained and analyzed using the Image-Pro Plus software program (version 4.5). Each well was observed, and the width of the wound was measured. (B) Effect of rLECT2 protein on VEGF-induced HUVEC tube formation. Tubular structures were quantified via manual counting in low-power fields (40×). (C) Effect of rLECT2 protein on VEGF-induced blood vessel formation in chick CAMs. (D) Effect of rLECT2 protein on VEGF-induced angiogenesis response in a Matrigel plug assay.


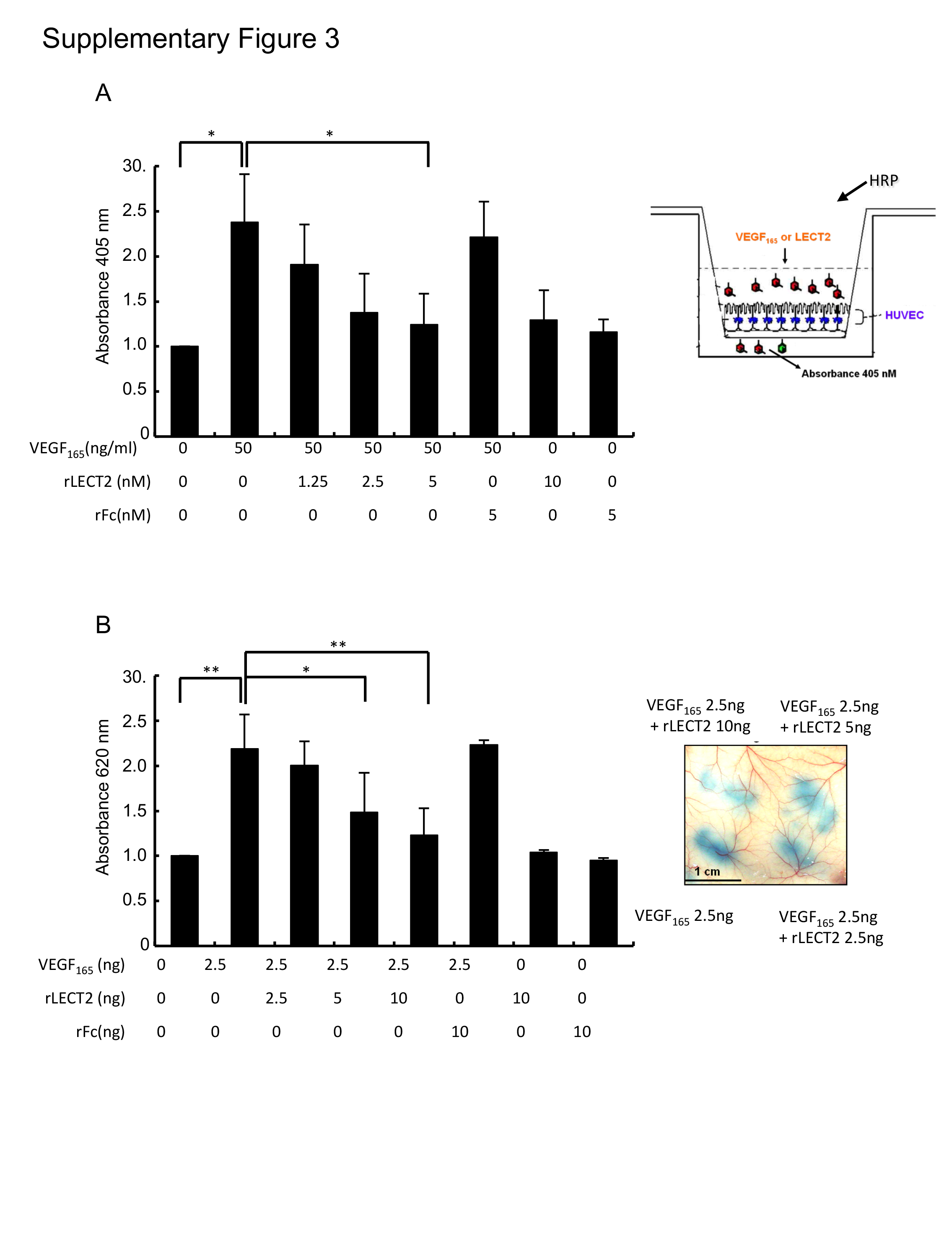
**Supplementary Fig. S3. rLECT2 protein regulates VEGF_165_-induced permeability *in vitro* and *in vivo*.** (A) Effect of rLECT2 protein expression on VEGF_165_-induced HUVEC permeability *in vitro*. A HUVEC monolayer was treated with VEGF_165_ with or without rLECT2 protein and Fc-Tag for the permeability assay described in supplementary experimental procedures. PBS rLECT2 protein or Fc-Tag alone were used as negative controls; whereas VEGF_165_ alone as negative control. The HUVEC permeability status in each group of treatment was measured according to the absorbance of extracted dye at 405 nm. (B) Effect of rLECT2 protein on VEGF_165_-induced HUVEC permeability *in vivo*. Evans blue dye (1 mg/ml) was injected into mice via the tail vein. Ten minutes later, 50 μl of VEGF_165_ (2.5 ng), VEGF**_165_** combined with rLECT2 protein (2.5-10.0 ng), or PBS was injected intradermally into back skin harvested from the mice. The absorbance of extracted dye was measured at 620 nm. Data are presented as the mean ± SD in triplicate. A paired Student *t*-test was used to evaluate statistically significant differences in blood vessels between treatment with VEGF_165_ alone and co-treatment of it with rLECT2 protein. **P* ＜ 0.05; ***P* ＜ 0.01.

**
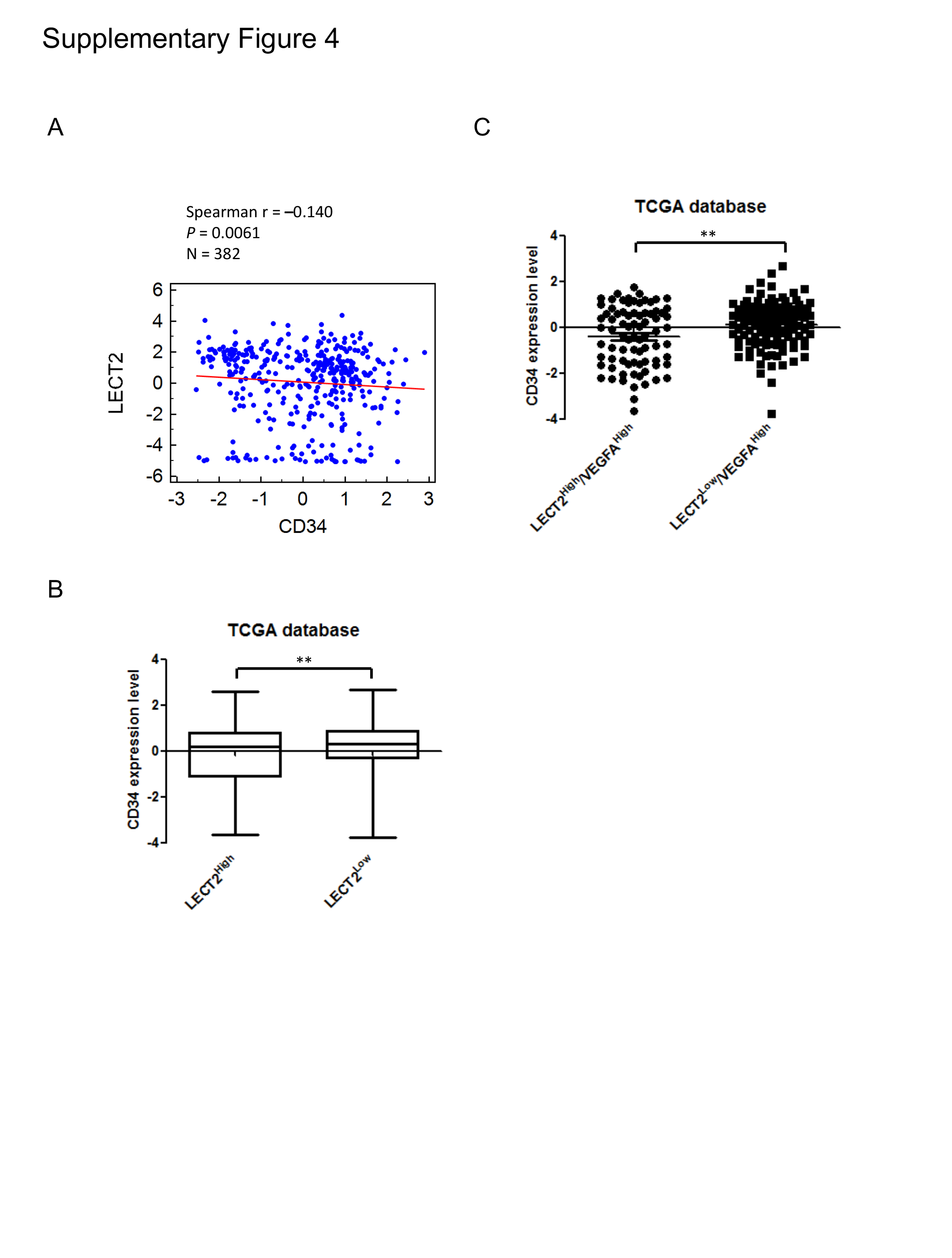
Supplementary Fig. S4. LECT2 expression is negatively correlated with angiogenesis in HCC patients according to data from The Cancer Genome Atlas (TCGA) database.** (A) Gene expression scatter diagram for LECT2 versus CD34 in HCC patients using data from The Cancer Genome Atlas database. The blue dots represent the expression levels in individual samples in the cohort. A regression line is shown on the plot. (B) Correlation between CD34 and LECT2 gene expression. Individual samples of HCC patients were grouped according to high and low LECT2 expression. (C) Correlation between CD34 and LECT2 with high VEGF_165_ gene expression.

**Supplementary Materials and Methods**

*Antibodies and reagents*

Recombinant human PDGF, EGF, and HGF protein were obtained from R&D Systems (Minneapolis, MN, USA) and recombinant human VEGF_165_ and bFGF protein from PeproTech (Rocky Hill, NJ, USA). Matrigel was obtained from BD Biosciences (Franklin Lakes, NJ, USA). Human phosphorylated p44/p42 mitogen-activated protein kinase (Thr202/Tyr204), phosphorylated AKT (Ser473), AKT, and pY99 antibodies were purchased from Santa Cruz Biotechnology (Santa Cruz, CA, USA); VEGFR2, phosphorylated p38, and p38 antibodies from Cell Signaling Technology (Danvers, MA, USA); CD34, CD31, and α-SMA from Dako (Carpinteria, CA, USA).

*rLECT2-rFc protein production and purification*

The p-EF-BOS-IgMu vector with *Bam*HI and *Not*I was used to construct LECT2, and oligonucleotides encoding for the LECT2 forward and reverse residues were generated (sense primer, 5'-TAATCAGC GGCCGCAACTAAAGGAAGTAAAACC-3'; antisense primer, 5'-GCCTTCGCTCGAGAGGTATGCAGTAGGGTC-3'). p-EF-BOS-IgMu-LECT2 plasmid was transfected into 293T cells and purified using protein agarose A. The size and purity of recombinant LECT2 were confirmed by Coomassie blue staining and separated on a sodium dodecyl sulfate-polyacrylamide gel electrophoresis gel.

*Transfection and established stable cell clones*

An LECT2 expression vector was constructed by placing human LECT2 cDNA in a pSecTag2A eukaryotic expression vector containing the hygromycin B gene under the control of the same promoter. LECT2 expression constructs were transfected into hepatoma cells using Lipofectamine 2000 reagent (Invitrogen, Carlsbad, CA, USA). Stable cell populations such as Skewere selected using 50 μg/ml hygromycin B, and single clones were confirmed to have prominent expression of LECT2 using reverse transcription-polymerase chain reaction and western blot analysis.

*Proliferation assay*

HUVECs undergoing 80% confluent growth were trypsinized, transferred to 24-well plates (2.5 × 10^4^ cells/well), and preincubated in EGM-2 medium for 24 h. The culture medium was then changed to starvation medium only, changed to starvation medium containing VEGF_165_ (50 ng/ml) or other indicated angiogenic factors, or combined with recombinant human LECT2 (rLECT2) protein at various concentrations and incubated at 37 °C for 48 h. At the end of this period, viable cell numbers were estimated using an MTT assay. Two hundred microliters of MTT solution (2 mg/ml; Sigma Chemical Co., St. Louis, MO, USA) was added to each well 2 h before experiment turnover, and the mixture was incubated in darkness. Absorbance was measured using an enzyme-linked immunosorbent assay reader (Bio-Rad, Hercules, CA, USA) at 570-nm absorbance. All experiments were carried out in triplicate, and the results are presented as the mean ± standard deviation (SD).

*Ex vivo* *chick chorioallantoic membrane assay*

Fertilized chicken eggs were incubated at 37 °C in 60% humidity and forced air circulation incubator for 20 days. On day 9, a small hole was drilled on the top of each egg above the area of greatest vasculature, causing the chorioallantoic membrane (CAM) to detach from the shell membrane. VEGF_165_ alone or treated with different concentrations of rLECT2 protein (0, 1.25, 2.50, or 5.00 nM) or control rFc protein in 5-mm-diameter discs and at a total volume of 20 μl were inoculated onto the dropped CAMs with a pipette tip (three eggs per group). The holes were then sealed with tape, and the eggs were returned to the incubator. The effect of rLECT2 on angiogenesis in the CAMs was quantitatively evaluated by scoring blood vessels in the control and treated groups. The blood vessels were scored using the Image-Pro Plus software program (version 4.5; MediaCybernetics).

*In vivo Matrigel plug assay*

VEGF_165_ alone or combined with rLECT2 protein (0, 1.25, 2.50, or 5.00 nM) was mixed with Matrigel (500 μl) and injected subcutaneously into 9-week-old female C57BL/6J mice (20 g) at sites lateral to the abdominal midline according to a protocol for the Matrigel plug assay . Animals were killed via CO_2_ asphyxiation 10 days after Matrigel injection, and the plugs were removed and photographed immediately. The plugs were then minced and homogenized with a tissue homogenizer, and absorbance was measured using an enzyme-linked immunosorbent assay reader. Hemoglobin levels in the Matrigel plugs were determined using Drabkin’s solution (Sigma Chemical Co.) according to the manufacturer’s instructions.

*Monolayer permeability assay*

Horseradish peroxidase (HPR) diffusion through a HUVEC monolayer was measured as described previously . HUVECs were cultured in Transwell chambers (0.4-μm-pore polycarbonate filters; Costar, Cambridge, MA, USA). After reaching confluence, cells were washed, and their medium was replaced with CM (150 μl in the upper chamber and 750 μl in the lower chamber). HPR molecular (0.126 μM Type VI-A, 44 kDa; Sigma, St. Louis, MO, USA) was added to the upper compartment, and the chambers were incubated at 37 °C. After 15 min, the medium in the lower compartment was assayed for HPR activity using a photometric guaiacol substrate assay according to the manufacturer’s instructions.

*Miles vascular permeability assay*

To determine the effect of Lect2 on vascular permeability *in vivo*, a modified Miles assay was performed as described below. Mice were given intravascular injections of 200 μl of Evans blue dye (0.5%) via the tail vain. Ten minutes later, mice received intradermal injections of 50 μl of PBS or recombinant human VEGF_165_ (50 ng/ml) or combination with rLECT2 (0, 1.25, 2.5, 5nM). The subdermis of injection site was harvested and photographed 30 min later to document any leakage of the dye into the dermal tissue.

*In vitro binding assay*

Binding of the His-tagged extracellular domain of VEGFR2 with Fc-tagged LECT2 was assessed according to Fc tag pull-down of the bound complexes with protein A-Sepharose. His-tagged recombinant VEGFR2 extracellular domain protein (Sino Biological Inc., North Wales, PA, USA) was mixed with Fc-tagged LECT2 in binding buffer (50 mM sodium phosphate, pH 7.5, 500 mM NaCl, 1% Nonidet P-40 in a final volume of 150 μl) and incubated at 4 °C for 4 h with gentle rotation. Protein A-Sepharose beads (50 μl) pre-equilibrated in binding buffer were added to the mixture, which was then incubated at 4 °C for 2 h with gentle rotation. The mixture was briefly centrifuged to sediment the resin beads, and the beads were washed three times with binding buffer. The proteins were eluted from the resin beads with 50 ml of 2X Laemmli buffer and analyzed in a Western blot against anti-LECT2 and anti-VEGFR2 antibodies.

*Western blotting*

Cells were lysed in RIPA buffer, and the protein concentration was measured using a Bradford protein assay. Proteins in the total cell lysate (40 μg of protein) were separated using sodium dodecyl sulfate-polyacrylamide gel electrophoresis in 10% gels and electrotransferred to polyvinylidene fluoride membranes (Millipore, Bedford, MA, USA). After the transferred membrane was blocked in a 5% skim milk solution, 0.1% Tween 20, and PBS, membrane-bound proteins were probed with indicated antibodies. The membrane was washed and incubated with horseradish peroxidase-conjugated secondary antibodies (Santa Cruz Biotechnology) for 30 min. Enhanced chemiluminescence reagents (Bio-Rad) were used to depict the protein bands on the membranes, which were developed using Kodak X-OMAT Blue autoradiography film (Eastman Kodak, Rochester, NY, USA).

*RTK phosphorylation array*

The expression of phosphorylated growth factors RTKs in HUVECs was detected using a Proteome Profiler Array Kit (R&D Systems). After 16 h of starvation, HUVECs were treated with rLECT2 protein or control Fc protein in EGM-2 medium for 20 min. The cells were collected in cold solubilization buffer (1% Triton X-100, 1 mM sodium vanadate, 1 mM sodium fluoride, 0.05 mM sodium molybdate, 20 μg/ml aprotinin, 20 μg/ml leupeptin, 4 μg/ml [4-amidinophenyl] methane sulfonyl fluoride, 150 mM sodium chloride in 50 mM Tris-HCl, pH 7.4). The cells were pipetted up and down for resuspension, and the cell lysates were rocked gently at 2­–8 °C for 30 min. The lysates were microcentrifuged at 14,000 × *g* for 5 min, and the supernatant was transferred into a clean test tube and stored at ­–80°C. Total protein (500 μg) from the HUVECs was incubated with RTK array membranes spotted with various anti-phospho-RTK antibodies. These procedures were performed according to the manufacturer’s protocol (R&D Systems).

**Supplementary References**

1. Malinda KM. In vivo matrigel migration and angiogenesis assay. Methods Mol Biol 2009;467:287-94.

2. Essler M, Retzer M, Bauer M, Heemskerk JW, Aepfelbacher M, Siess W. Mildly oxidized low density lipoprotein induces contraction of human endothelial cells through activation of Rho/Rho kinase and inhibition of myosin light chain phosphatase. J Biol Chem 1999;274(43):30361-4.

3. Murohara T, Horowitz JR, Silver M, Tsurumi Y, Chen D, Sullivan A, et al. Vascular endothelial growth factor/vascular permeability factor enhances vascular permeability via nitric oxide and prostacyclin. Circulation 1998;97(1):99-107.
